# Novel Aspects of Nitrate Regulation in *Arabidopsis*

**DOI:** 10.3389/fpls.2020.574246

**Published:** 2020-12-10

**Authors:** Hongmei Fan, Shuxuan Quan, Shengdong Qi, Na Xu, Yong Wang

**Affiliations:** ^1^State Key Laboratory of Crop Biology, College of Life Sciences, Shandong Agricultural University, Tai’an, China; ^2^School of Biological Science, Jining Medical University, Rizhao, China

**Keywords:** nitrate regulation, leaf senescence, circadian clock, post-transcriptional regulation, non-coding RNA

## Abstract

Nitrogen (N) is one of the most essential macronutrients for plant growth and development. Nitrate (NO_3_^–^), the major form of N that plants uptake from the soil, acts as an important signaling molecule in addition to its nutritional function. Over the past decade, significant progress has been made in identifying new components involved in NO_3_^–^ regulation and starting to unravel the NO_3_^–^ regulatory network. Great reviews have been made recently by scientists on the key regulators in NO_3_^–^ signaling, NO_3_^–^ effects on plant development, and its crosstalk with phosphorus (P), potassium (K), hormones, and calcium signaling. However, several novel aspects of NO_3_^–^ regulation have not been previously reviewed in detail. Here, we mainly focused on the recent advances of post-transcriptional regulation and non-coding RNA (ncRNAs) in NO_3_^–^ signaling, and NO_3_^–^ regulation on leaf senescence and the circadian clock. It will help us to extend the general picture of NO_3_^–^ regulation and provide a basis for further exploration of NO_3_^–^ regulatory network.

## Introduction

Nitrogen (N) is a macronutrient required by many important biological processes in plants and also a limiting factor for crop production in the agricultural system. Most crops such as maize and wheat absorb nitrate (NO_3_^–^) as the main N forms. NO_3_^–^ uptake, transport, and assimilation are the principal processes for NO_3_^–^ utilization and have long been studied. Besides its nutritional role, NO_3_^–^ can also serve as a signaling molecule that modulates plant growth and development. During the last decade, great progress has been made on NO_3_^–^ regulation in root system architecture, shoot growth, seed dormancy, flowering time, as well as crosstalk with other signals (hormone, calcium) and nutrients (P and K) (Footitt et al., 2011, 2013; Bouguyon et al., 2015, 2016; Riveras et al., 2015; Yan et al., 2016; Yuan et al., 2016; Li et al., 2017; Lin and Tsay, 2017; Gaudinier et al., 2018; Gras et al., 2018; Du et al., 2019; Hu et al., 2019; Medici et al., 2019). Some crucial components involved in NO_3_^–^ signaling have been identified, such as ANR1, NRT1.1, NLP6/7, TCP20, LBD37/38/39, TGA1/4, SPL9, bZIP1, NAC4, NRG2, and NIGT/HRS1 (Zhang and Forde, 1998; Remans et al., 2006; Ho et al., 2009; Rubin et al., 2009; Krouk et al., 2010; Konishi and Yanagisawa, 2013; Marchive et al., 2013; Alvarez et al., 2014; Guan et al., 2014; Para et al., 2014; Vidal et al., 2014; Xu et al., 2016; Cao et al., 2017; Kiba et al., 2018; Maeda et al., 2018; Sun et al., 2018a, b; Zhao et al., 2018). These progress have been summarized in several excellent review papers (Gutiérrez, 2012; O’Brien et al., 2016; Mu and Luo, 2019; Jia and von Wirén, 2020; Liu et al., 2020; Luo et al., 2020; Vidal et al., 2020). However, the research on nitrate regulation at post-transcriptional levels is still lacking. Using nitrogen use efficiency (NUE) genes to breed high NUE cultivars is essential to sustain crop productivity. Several genes regulating NUE have been characterized in crops, such as *OsNRT1.1B*, *OsNRT2.1*, *OsNRT2.3b*, *OsBT1/2*, *OsGRF4*, *OsDEP1, OsGCR1, OsGPA1, OsNAC42*, *ZmNAC7*, *TaNFYA*, *TaDOF1* (Sun et al., 2014; Chakraborty et al., 2015, 2019; Hu et al., 2015; Qu et al., 2015; Araus et al., 2016; Chen M. et al., 2016; Fan et al., 2017; Peña et al., 2017; Li et al., 2018; Liang et al., 2018; Sharma et al., 2018; Tang et al., 2019; Zhang et al., 2019; Pathak et al., 2020), which have also been well reviewed (Wang et al., 2018a; Zuluaga and Sonnante, 2019; Vidal et al., 2020).

Following the application of new technologies like multi-omics, post-transcriptional regulators of NO_3_^–^ including the mRNA splicing factors CPSF30-L, FIP1, and non-coding RNAs (ncRNAs) have been characterized as new players in NO_3_^–^ signaling (Zhao et al., 2011; Li et al., 2017; Wang et al., 2018b; Liu et al., 2019). In addition, novel functions and mechanisms of NO_3_^–^ on plant development have been revealed. Scientists found that *NRT1.5* and *NLA* (*Nitrogen Limitation Adaptation*) could repress N deficiency-induced leaf senescence (Meng et al., 2016; Liu et al., 2017). Regarding the effects of N on circadian clock, the center clock genes *CCA1*, *LHY*, and *TOC1* have been found to play important roles (Gutiérrez et al., 2008; Yuan et al., 2016; Teng et al., 2017). In this review, we mainly focus on these advances in NO_3_^–^ regulation. It will provide more insights to better understand the complexity and mechanism of the NO_3_^–^ regulation and lay a fundamental base for further deciphering the NO_3_^–^ regulatory network and improving NUE in agriculture.

### Post-transcriptional Regulation of Nitrate

The importance of post-transcriptional modifications of genes associating with a wide range of plant responses has been already described in plants (Kim et al., 2008; Kwon et al., 2009; Berr et al., 2010; Nelissen et al., 2010). However, the role of post-transcriptional modifications in N regulation is still poorly understood. The factor IWS1 has been shown to be involved in several aspects of transcription, like transcription elongation, recruitment of chromatin-remodeling factors, mRNA processing and export, and post-transcriptional histone modification (Krogan et al., 2002; Yoh et al., 2007, 2008; Zhang et al., 2008). Lepetit’s group found that *IWS1* represses *NRT2.1* transcription in response to high N supply, which is associated with an IWS1-dependent accumulation of histone H3 lysine 27 trimethylation (H3K27me3) (Widiez et al., 2011). The result suggests that *IWS1* represses gene expression by specifically promoting H3K27me3 methylation under high N conditions. This is the first evidence demonstrating that post-transcriptional chromatin modifications regulate N acquisition. More recently, Coruzzi’s group reported that histone methyltransferase SET DOMAIN GROUP8 (SDG8) mediates genome-wide changes of H3K36me3 after NO_3_^–^ treatments, resulting in the altered expression of its target genes, RNA processing, and physiological responses (Li et al., 2020). More epigenetic marks, such as other histone modifications (e.g., histone acetylation), chromatin landscape, and DNA methylation need to be investigated in the future to gain a comprehensive understanding of chromatin regulation in NO_3_^–^ signaling.

RNA processing is important for the post-transcriptional regulation of gene expression, and is a rate-limiting step in the expression of proteins. The *CPSF30* gene encodes the cleavage and polyadenylation specificity factor, which forms a larger protein (CPSF30-L) with 65 kDa and a smaller protein (CPSF30-S) with 28 kDa. Both proteins possess three characteristic C3H zinc finger motifs and act as an RNA-binding protein and an endonuclease (Addepalli and Hunt, 2007). The CPSF30-L contains an additional YT512-B Homology (YTH) domain along with the three zinc finger motifs (Li et al., 2017). By using forward genetics, the *CPSF30-L* gene was cloned and found that it plays a crucial role in NO_3_^–^ signaling, transport, and assimilation in *Arabidopsis* (Li et al., 2017). Molecular and genetic assays revealed that *CPSF30-L* works upstream and regulates the expression of *NRT1.1*, while it functions independently of *NLP7* in NO_3_^–^ signaling. FIP1, another RNA-binding protein, is also a core component of a pre-mRNA processing complex (Forbes et al., 2006). Further research showed that FIP1 interacts with CPSF30-L and regulates NO_3_^–^ signaling and assimilation (Wang et al., 2018b). Both *CPSF30-L* and *FIP1* modulate the expression of NO_3_^–^ regulatory genes *CIPK8* and *CIPK23* and influence the alternative polyadenylation (APA) of the 3′-UTR of *NRT1.1* mRNA (Li et al., 2017; Wang et al., 2018b). These results indicate that *CPSF30-L* and *FIP1* may modulate NO_3_^–^ signaling through influencing the APA processing of *NRT1.1*.

RNA can be chemically modified with many different reactions, among which methylation is one of the most well studied modification. N^6^-methyladenosine (m^6^A) is a pivotal internal mRNA modification, which plays crucial roles in plant growth and development by regulating gene expression at the post-transcriptional level. Recently, it has been reported that the EVOLUTIONARILY CONSERVED C-TERMINAL REGION2 (ECT2) acts as an m^6^A reader depending on the presence of the YTH domain (Wei et al., 2018; Yue et al., 2019). As CPSF30-L contains an YTH domain, whether it also serves as the m^6^A reader to regulate NO_3_^–^ signaling needs further investigation.

There are over 100 types of RNA modifications (Roundtree et al., 2017; Bailey-Serres et al., 2020), yet our knowledge about their occurrence and functions are still limited. One thing is certain though: post-transcriptional regulation of gene expression is much more intricate than previously thought. Unraveling the functions and underlying mechanisms of RNA modifications are essential to understand the roles of NO_3_^–^ regulatory genes and will provide valuable insights into post-transcriptional regulation of NO_3_^–^.

### Nitrate and ncRNAs

**FIGURE 1:**
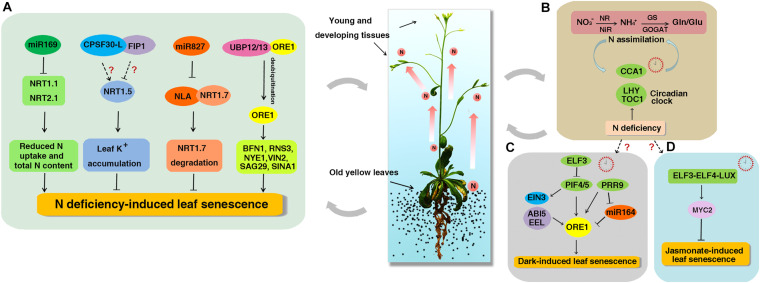
The working model of NO_3_^−^ regulation on leaf senescence and circadian clock. Leaf senescence accompany with N reallocation to meet the demand of N in young and developing tissues. **(A)** The miR169 reduces the N uptake and total N content by decreasing the expression of *NRT1.1* and *NRT2.1* and promotes the early senescence induced by N starvation. *NRT1.5* increases foliar K^+^ levels to suppress NO_3_^−^ starvation-induced leaf senescence. *CPSF30-L* interacts with *FIP1* and both can regulate the expression of *NRT1.5*, but if both genes participate in NO_3_^−^ starvation-induced leaf senescence remains unknown. As a target of miR827, NLA directly interacts with *NRT1.7* and promotes its degradation to repress leaf senescence. UBP12 and UBP13 interact with ORE1 and promote its deubiquitination to prevent ORE1 from degradation. The increased ORE1 levels activate the expression of downstream SAGs such as *BFN1*, *RNS3*, *NYE1*, *VNI2*, *SAG29*, and *SINA1* to promote N deficiency-induced leaf senescence. **(B)** A feedback loop between N assimilation and circadian clock. Organic N influences the *CCA1* phase and *CCA1* in turn regulates the expression of organic N-regulated genes. N deficiency condition can increase the amplitudes of *CCA1*, *LHY*, and *TOC1* transcripts. **(C)** ELF3 inhibits the expression of *PIF4/PIF5* to repress dark-induced leaf senescence. PIF4 and PIF5 promote dark-induced senescence by directly activating the expression of *ORE1* and indirectly activating the expression of *EIN3, ABI5*, and *EEL*. PRR9 can activate the expression of *ORE1* directly or suppress the expression of miR164 to promote dark-induced leaf senescence. **(D)** The circadian evening complex (ELF3-ELF4-LUX) directly binds to the promoter of *MYC2* to repress jasmonate-induced leaf senescence. However, if circadian clock is involved in N deficiency-induced senescence remains unknown. The solid and dotted arrows represent known and unknown functions, respectively.

Whole-genome sequencing analyses have identified thousands of ncRNAs playing vital roles in numerous biological processes in plants (Laporte et al., 2007; Rymarquis et al., 2008). Several microRNAs (miRNAs) and a long ncRNA (lncRNA) have been reported to function in NO_3_^–^ regulation in *Arabidopsis* and crops (Miin-Feng et al., 2006; Vidal et al., 2010; Liu et al., 2019; Table 1). In *Arabidopsis*, miR167 targets two auxin responsive factors ARF6 and ARF8. The miR167/ARF8 module regulates lateral root growth in response to NO_3_^–^ by controlling a group of NO_3_^–^-responsive genes (Miin-Feng et al., 2006; Gifford et al., 2008). The miR393 and AFB3 can respond to NO_3_^–^ by integrating internal organic N signals, external NO_3_^–^ availability and root auxin sensitivity to control root architecture (Vidal et al., 2010). Upon N starvation, the expression of miR160, miR164, miR167, miR780, miR826, miR842, miR846, and miR5090 is induced, whereas the expression of miR169, miR171, miR395, miR397, miR398, miR399, miR408, miR827, and miR857 is repressed (Xu et al., 2011; Liang et al., 2012; He et al., 2014; Li et al., 2016). Among them, miR160, miR167, and miR171 can be responsible for the development of root systems under N-starvation conditions (Liang et al., 2012). Moreover, miR826 and miR5090 suppress the expression of *ALKENYL HYDROXALKYL PRODUCING 2* (*AOP2*), which is involved in the modification of the glucosinolate side chain. Overexpression of miR826 and miR5090 results in less glucosinolate and enhanced tolerance to N starvation (He et al., 2014). Overexpression of miR169 reduces N uptake and total N content by decreasing the expression of *NRT1.1* and *NRT2.1* and thus promotes N starvation-induced early leaf senescence (Zhao et al., 2011; Figure 1A). In addition, the miR5640 post-transcriptionally regulates the expression of its target *AtPPC3* in response to NO_3_^–^ (Vidal et al., 2013). The N-responsive miRNA miR156 works upstream of SPL9, a SBP-box transcription factor (TF) identified to control the genes involved in the NO_3_^–^ primary response (Krouk et al., 2010), implying that the miR156/SPL9 module functions in NO_3_^–^ regulation. MiRNAs have also been found to be responsive to N limitation in crops (Xu et al., 2011; Trevisan et al., 2012). MiR169 is up-regulated in maize but down-regulated in rice under N starvation condition (Xu et al., 2011; Li et al., 2016). Two novel putative miR169 species miRC10 and miRC68 are reported to play major roles in the adaptation to NO_3_^–^ limitation in maize seedlings (Zhao et al., 2013). Furthermore, OsmiR444a plays multiple roles including NO_3_^–^-dependent root growth, NO_3_^–^ accumulation and phosphate-starvation responses in rice (Yan et al., 2014). Chengcai Chu’s group reported that OsmiR3979 functions as a key regulator to optimize root growth in response to NO_3_^–^ (Li et al., 2016). Moreover, OsmiR159a.1 can be strongly repressed by ammonium, which is opposite to its response to NO_3_^–^. It targets *LOC_Os06g40330* and *LOC_Os01g59660*, encoding MYB family TFs, and cleaves them at the predicted cleavage sites (Jeong et al., 2011; Li et al., 2016). Those results suggest that miRNAs play critical roles in NO_3_^–^ regulation by modulating the expression of their target genes.

**TABLE 1:** Characterized ncRNAs involved in NO_3_^–^ regulation.

Gene name	Targets	Species	Reference
miR156	*SPL9*	*Arabidopsis*	Krouk et al., 2010
miR160	Auxin response factors	*Arabidopsis*	Liang et al., 2012
miR167	*ARF6*, *ARF8*	*Arabidopsis*	Miin-Feng et al., 2006
miR169	CCAAT-binding transcription factor	*Arabidopsis*, Maize, Rice	Zhao et al., 2011
miR171	SCL transcription factors	*Arabidopsis*	Liang et al., 2012
miR393	*AFB3*	*Arabidopsis*	Vidal et al., 2010
miR5090	*AOP2*	*Arabidopsis*	He et al., 2014
miR5640	*PPC3*	*Arabidopsis*	Vidal et al., 2013
miR826	*AOP2*	*Arabidopsis*	He et al., 2014
miR827	*NLA*	*Arabidopsis*, Maize	Liu et al., 2017
miR846	Jacalin lectin family	*Arabidopsis*	Xu et al., 2011
miR444a	MIKC-type MADS-box genes	Rice	Yan et al., 2014
miR3979	*IAA3*	Rice	Li et al., 2016
miR159a.1	*LOC_Os06g40330 LOC_Os01g59660*	Rice	Jeong et al., 2011; Li et al., 2016
lncRNA *T5120*	Unknown	*Arabidopsis*	Liu et al., 2019

Long non-coding RNAs are a class of ncRNAs with a length longer than 200 nucleotides and poor protein-coding potential (Pang et al., 2006; Ponting et al., 2009). A multitude of lncRNAs have been identified by using next-generation sequencing during the last several years, but only a few have been characterized (Xin et al., 2011; Liu et al., 2012; Meng et al., 2012). It has been reported that lncRNAs play crucial roles in flowering, phosphate starvation, polar auxin transport, photomorphogenesis, mRNA alternative splicing, and plant immune response (Francozorrilla et al., 2007; Amor et al., 2009; Swiezewski et al., 2009; Heo and Sung, 2011; Bardou et al., 2014; Seo et al., 2017). Several papers described changes of lncRNA responding to N treatments or N deficiency in *Populus* and maize (Vidal et al., 2013; Alvarez et al., 2014; Chen M. et al., 2016; Lv et al., 2016). However, the role of lncRNAs in N regulation remains largely unclear. Our group found six NO_3_^–^-induced lncRNAs using RNA-seq technology and validated by qRT-PCR. Further investigation revealed that the *TCONS_00005120* (*T5120*), showing the highest induction by NO_3_^–^, could regulate NO_3_^–^ response and assimilation. Molecular and genetic assays showed that *NLP7* directly binds to the promoter of *T5120* to regulate its expression. In addition, the expression of *T5120* was modulated by *NRT1.1* (Liu et al., 2019). This is the first attempt to investigate the role of lncRNAs in NO_3_^–^ signaling and provides a fundamental base for discovering more lncRNAs functioning in NO_3_^–^ regulation.

### Nitrate and Leaf Senescence

Leaf senescence is a developmental process accompanying resource rearrangement (Havé et al., 2017). It occurs in a coordinated manner, starting with the inhibition of leaf expansion, and then the induction of metabolic changes followed by degradation of organic substances and nutrient remobilization (Soltabayeva et al., 2018). Some environmental stresses can induce early leaf senescence, among which N deficiency tends to promote leaf senescence to meet the demand of N in young and developing tissues (Aguera et al., 2010).

Although it is previously known that N deficiency can accelerate leaf senescence (Schildhauer et al., 2008; Gregersen et al., 2013), the underlying mechanisms are still poorly understood. It has been found that *NRT1.5* functions in transporting NO_3_^–^ from the roots to the shoots (Lin et al., 2008) and the expression of *NRT1.5* is strongly induced by leaf senescence in *Arabidopsis* (Van Der Graaff et al., 2006). Jiming Gong’s lab has recently reported that *NRT1.5* suppresses the leaf senescence induced by NO_3_^–^ (but not nitrogen) starvation (Meng et al., 2016) and this suppression is independent of the NO_3_^–^ transport function of *NRT1.5*. Further analyses showed that foliar K^+^ levels decreased in *nrt1.5* when exposed to NO_3_^–^ starvation and adding K^+^ could restore the early leaf senescence phenotype of *nrt1.5* plants, suggesting that *NRT1.5* may increase foliar K^+^ levels to suppress NO_3_^–^ starvation-induced leaf senescence (Meng et al., 2016; Figure 1A). Lately, it was found that the expression of *NRT1.5* could be positively modulated by *CPSF30-L* (Li et al., 2017) and suppressed by *FIP1* (Wang et al., 2018b). Therefore, it would be interesting to investigate if both genes participate in NO_3_^–^ starvation-induced leaf senescence (Figure 1A).

*Nitrogen limitation adaptation* encodes a RING E3 ubiquitin ligase. A previous study showed that the *nla* mutant could not accumulate anthocyanin and instead exhibited early senescence phenotype under N deficiency condition (Peng et al., 2008). Recently, Wenxue Li’s group confirmed this result and found that the content of ^15^N in the young leaves of *nla* mutant is significantly higher than that of wild-type (WT), indicating that NLA decreases translocation of N from the old leaves to the young leaves (Liu et al., 2017). It has been reported that *NRT1.7* works in transporting NO_3_^–^ from the old to young leaves as less ^15^NO_3_ spotted onto old leaves is remobilized to young leaves in *nrt1.7* mutant compared with WT (Fan et al., 2009). Further investigation demonstrated that NLA directly interacts with *NRT1.7* and promotes *NRT1.7* degradation *in vivo* to repress early senescence. The *NLA* gene is a target of miRNA827. N limitation strongly repressed the NLA protein abundances in WT, while the loss of function of miRNA827 abolished the repression of NLA protein and down-regulated the *NRT1.7* protein abundance under N limitation condition, indicating that N deficiency-induced repression of NLA is dependent on miRNA827 (Liu et al., 2017; Figure 1A).

An extensive reprogramming of gene expression has been found to be involved in leaf senescence to meet the complex biochemical and structural changes. Up to now, about 100 TFs that belong to about 20 different families (in particular, bZIP, WRKY, NAC, C2H2 zinc finger, MYB, and AP2-EREBP) have been described to play important roles in leaf senescence (Guo et al., 2004; Buchanan-Wollaston et al., 2005; Ay et al., 2014). ORESARA1 (ORE1, also known as NAC92) is a key factor that promotes age-dependent leaf senescence by regulating the expression of hundreds of senescence-associated genes (SAGs). These SAGs function mainly in the breakdown of nucleic acids and proteins, and the transport of sugar in *Arabidopsis* (Balazadeh et al., 2010a; Matallana-Ramirez et al., 2013). Nam-Hai Chua’s group showed that ORE1 interacts with NLA and UBP12/13 (ubiquitin-specific protease 12/13) to regulate leaf senescence (Park et al., 2014, 2018, 2019). Under N sufficient condition, ORE1 is polyubiquitinated by NLA and its E2 conjugase PHO2 (UBC24), and then degraded by 26S proteasomes leading to delayed leaf senescence (Park et al., 2018). But when plants were exposed to N-deficient conditions, UBP12/UBP13 antagonize the action of NLA to maintain ORE1 homeostasis by promoting the deubiquitination of ORE1 (Park et al., 2019). The elevated ORE1 levels activate the expression of downstream SAGs such as *BFN1*, *RNS3*, *NYE1*, *VNI2*, *SAG29*, and *SINA1* resulting in early leaf senescence (Park et al., 2019; Figure 1A). Those results provide us the post-translational regulation of N deficiency-induced leaf senescence. In addition, miR164 regulates age-induced leaf senescence by targeting *ORE1* mRNA cleavage (Kim et al., 2009; Balazadeh et al., 2010b). But miR164 does not regulate the expression of *ORE1* at transcriptional levels under N-starvation condition (Liang et al., 2015; Park et al., 2018).

HY5 is a key regulator of photomorphogenesis (Lee et al., 2007). About 30% of the senescence-related regulatory genes are predicted as putative targets of the *HY5*, such as *AP2-EREBP*, *NAC*, and *WRKY* (Breeze et al., 2011; Ay et al., 2014), suggesting that *HY5* may play an important role in the regulatory network of leaf senescence. Interestingly, HY5 has been found to activate the expression of *NIA1*, *NIA2*, and *NRT2.1*, but inhibit the expression of *NRT1.1* (Jonassen et al., 2009a, b; Chen X. et al., 2016), implying that HY5 may be involved in NO_3_^–^ regulation. Recently, the light-signaling protein FAR-RED ELONGATED HYPOCOTYL3 (FHY3) was reported to negatively regulate salicylic acid (SA) biosynthesis and light-mediated leaf senescence (Tian et al., 2020). Both the *fhy3* mutant and *WRKY28*-overexpressing plants exhibited early leaf senescence under high (Red/Far-Red) R: FR light conditions. FHY3 could directly bind to the promoter of *WRKY28* to repress its expression. These results indicate that the FHY3–WRKY28 module prevents leaf senescence under high R: FR light conditions (Tian et al., 2020). It was reported previously that FHY3 and HY5 physically interact with each other and co-regulate the expression of *CONSTITUTIVE PHOTOMORPHOGENESIS1* (*COP1*), a multifunctional E3 ubiquitin ligase, in response to photomorphogenic UV-B (Huang et al., 2012). Further investigation is needed to determine if FHY3, COP1, and HY5 function in N starvation-induced leaf senescence.

Although N starvation induces senescence in plants, it has also been reported that senescence is reversible in some cases after the resupply of N (Schildhauer et al., 2008). Mueller-Roeber’s group further performed transcriptome analysis to investigate the N resupply induced reversal of senescence in *Arabidopsis*. Their results showed that the senescence program was tuned by the N status, indicating that plants undergoing senescence retain the capacity to sense and respond to the N availability (Balazadeh et al., 2014). Crops such as maize (Girardin et al., 1985), rice (Wang et al., 2012), or barley (Schildhauer et al., 2008), have also been found to have the ability to stop or even reverse the senescence induced by N deficiency when sufficient N is resupplied. However, the underlying mechanism still remains unclear. It is nevertheless evolutionarily important to have a regulatory system capable of monitoring and integrating such environmental stimuli.

### Nitrate and Circadian Clock

The circadian clock is one of the most central endogenous factors that allow organisms to synchronize internal biological activities with the external environment. There is increasing evidence that circadian clock regulates N assimilation (Tucker et al., 2004; Cookson et al., 2005; Gutiérrez et al., 2008; Teng et al., 2017). It has been shown that organic N such as Glu or Gln can serve as signals to regulate the expression of N-related genes in plants (Oliveira and Coruzzi, 1999). Coruzzi’s group performed a transcriptome analysis on plants treated with organic N (Gutiérrez et al., 2008). By using systems biology, they found that the center clock gene *CCA1* could regulate the expression of organic N-regulated genes. Further investigation revealed that CCA1 could directly bind to the promoters of the glutamine synthetase gene (*GLN1.3*) and the glutamate dehydrogenase gene (*GDH1*) to regulate their expression. Moreover, the phase response curve analysis exhibited that organic N could influence the *CCA1* phase. These data indicate that organic N may regulate circadian rhythms through CCA1-mediated mechanism (Gutiérrez et al., 2008). Another group found that low N conditions increased the amplitudes of *CCA1*, *LHY*, and *TOC1* transcripts throughout the circadian cycle, whereas high N conditions decreased the amplitudes of these genes in WT plants (Yuan et al., 2016). Further study showed that CCA1 influence nitrate reductase activity by binding to the CCA1-binding site in the promoters of *NIA1* and *NIA2 in vitro* and activating the *NIA1* expression while repressing *NIA2* expression (Teng et al., 2017). These results provide basic insight for the link between N and circadian clock (Figure 1B).

It has been reported that TCP20 binds to the promoters of *NIA1*, *NRT1.1*, *NRT2.1* and acts in systemic NO_3_^–^ signaling that directs NO_3_^–^ foraging in *Arabidopsis* roots (Guan et al., 2014). Furthermore, TCP20 can physically interact with NLP6/7 and ARF8, and their interactions are required for activating the expression of the G2/M cell-cycle marker gene *CYCB1;1* to regulate lateral root initiation and growth (Guan et al., 2017; Fan et al., 2019a, b). Interestingly, TCP20 has also been found to be a circadian clock factor that can interact with LIGHT-REGULATED WD1 (LWD1). Both TCP20 and LWD1 can bind to the promoter of *CCA1* to activate its expression (Wu et al., 2016). These results imply that TCP20 may be involved in connecting the N signaling with circadian clock.

During last several years, scientists have demonstrated the roles of the circadian clock genes in the regulation of leaf senescence in plants (Sakuraba et al., 2014; Song et al., 2014; Kim et al., 2018; Zhang et al., 2018). PIF4/PIF5 promote dark-induced leaf senescence by directly activating the expression of *ORE1* and indirectly activating the expression of ETHYLENE INSENSITIVE 3 (*EIN3*) in ethylene signaling and bZIP factors *ABA INSENSITIVE 5* (*ABI5*) and *ENHANCED EM LEVEL* (*EEL*) in ABA signaling (Sakuraba et al., 2014; Song et al., 2014). ELF3 inhibits leaf senescence by repressing the activity of PIF4/PIF5 (Sakuraba et al., 2014; Figure 1C). The transcript levels of *ORE1* and its repressor miR164 show circadian rhythmic patterns (Kim et al., 2018). PRR9 can activate the expression of *ORE1* directly or suppress the expression of miR164 to promote dark-induced leaf senescence (Kim et al., 2018; Figure 1C). In addition, *elf4* and *lux* show obviously early leaf senescence than WT in darkness (Sakuraba et al., 2014; Kim et al., 2018). The circadian evening complex (ELF3-ELF4-LUX) can repress jasmonate-induced leaf senescence by directly binding to the promoter of *MYC2*, a key activator of jasmonate-induced leaf senescence (Zhang et al., 2018; Figure 1D). Ubiquitin-specific protease genes *UBP12* and *UBP13* are regulated by circadian clock and they in turn regulate circadian clock and photoperiodic flowering through *GI* and *CO*, which extends our understanding of deubiquitination in circadian clock and photoperiodic flowering regulation at posttranslational level (Cui et al., 2013). These results demonstrate a strong interplay between the circadian clock and leaf senescence. However, if circadian clock is involved in N deficiency-induced senescence remains unknown.

## Conclusions and Perspectives

During last several years, research on NO_3_^–^ regulation has expanded to multiple regulatory levels such as post-transcriptional modulations and also to some new developmental processes such as leaf senescence and circadian clock. The progress on these fields has started to shed light on better understanding the complex NO_3_^–^ regulatory network. However, we still lack an integrated view on how NO_3_^–^ regulates these processes and the full picture of NO_3_^–^ regulatory network is far from complete.

Post-transcriptional regulation of NO_3_^–^ has attracted more attention recently and gained further insights into NO_3_^–^ regulation. The C3H-type zinc finger motif-containing protein CPSF30-L has been characterized to function in NO_3_^–^ signaling (Li et al., 2017). The C3H-type zinc finger protein family contains 68 members and whether other members of this family participate in NO_3_^–^ regulation needs to be investigated. Although ncRNAs have been found to be involved in the regulation of multiple biological processes, the underlying mechanisms still remain elusive, especially in NO_3_^–^ signaling. The leaf senescence is finely tuned by N status. However, our understanding of the mechanisms that N availability affects leaf senescence is fragmentary and more efforts are still needed. One interesting avenue for future research is to screen mutants without early senescence symptoms under N-deficient conditions to identify the key components. In addition, systems biology may be used to globally reveal novel regulatory genes involved in N deficiency-induced leaf senescence. The core circadian genes *CCA1*, *LHY*, *TOC1* are the main factors that connect circadian clock and NO_3_^–^ regulation reported so far. Further studies are needed to find out more genes involved in circadian clock and NO_3_^–^ regulation and explore their intrinsic mechanisms. The application of translatomics, structural biology and other new technologies will surely promote the clarification of the mechanisms that NO_3_^–^ regulates plant development. Notably, the major part of the results therein resumed have been obtained in *Arabidopsis*, which although being an excellent model for studying NO_3_^–^ signaling and providing useful fundamental knowledge for the scientific community, is quite different from crops (especially monocots), for which specific and targeted research projects are required. With the identification of more novel NO_3_^–^ regulators and decryption of NO_3_^–^ regulatory networks, as well as combined with scientific and rational fertilization management, we will effectively improve NUE and reduce the environmental pollution caused by loss of N fertilizers.

## Author Contributions

HF and YW wrote the manuscript. SXQ, SDQ, and NX provided assistance for further modification of manuscript. All authors contributed to the article and approved the submitted version.

## Conflict of Interest

The authors declare that the research was conducted in the absence of any commercial or financial relationships that could be construed as a potential conflict of interest.

## Abbreviations

N: nitrogen
NO_3_^–^: nitrate
P: phosphorus
K: potassium
NUE: nitrogen use efficiency
WT: wild-type
T5120: TCONS_00005120
AOP2: ALKENYL HYDROXALKYL PRODUCING 2
NLA: nitrogen limitation adaptation
ORE1: ORESARA1
SAGs: senescence-associated genes
UBP12/13: ubiquitin-specific protease 12/13
FHY3: far-red elongated hypocotyl 3
SA: salicylic acid
COP1: constitutive photomorphogenesis1
EIN3: ETHYLENE INSENSITIVE 3
ABI5: ABA INSENSITIVE 5
EEL: ENHANCED EM LEVEL
GLN1.3: glutamine synthetase
GDH1: glutamate dehydrogenase
LWD1: LIGHT-REGULATED WD1
H3K27me3: histone H3 lysine 27 trimethylation
YTH: YT512-B Homology
ECT2: EVOLUTIONARILY CONSERVED C-TERMINAL REGION2
APA: alternative polyadenylation
m^6^A: N^6^-methyladenosine
miRNAs: microRNAs
ncRNAs: non-coding RNAs
lncRNAs: long non-coding RNAs.
